# Prevalence of Burnout Syndrome Among Saudi Emergency Department Physicians in Governmental General Hospitals in Jeddah, Saudi Arabia

**DOI:** 10.7759/cureus.90757

**Published:** 2025-08-22

**Authors:** Abdullah Abdulhameed S Alghamdi, Khalid Mohammed Alzahrani

**Affiliations:** 1 Preventive Medicine Department, General Directorate of Health Affairs, Jeddah, SAU

**Keywords:** burnout, depersonalization, emergency physicians, emotional exhaustion, personal achievement, saudi arabia

## Abstract

Background

Emergency department (ED) physicians are vulnerable to burnout due to the high-intensity nature of their work environment. However, studies focusing on emergency physicians in Saudi Arabia remain limited. This study aimed to determine the prevalence and risk factors of burnout syndrome among the ED physicians working in the governmental general hospitals in Jeddah, Saudi Arabia.

Methods

This cross-sectional study utilized an online, self-structured questionnaire to collect data from ED physicians in governmental hospitals in Jeddah, Saudi Arabia. The questionnaire included three sections: a) information about the study and consent, b) demographic data and work-related information, and c) the Maslach Burnout Inventory (MBI) with its three components (personal accomplishment (AP), depersonalization (DP), and emotional exhaustion (EE)). For the purposes of this study, the prevalence of burnout was calculated as the percentage of physicians with high-level burnout in at least one subscale.

Results

High-level burnout was detected in 66% of physicians (n = 66, 95% CI: 55.8%-75%) in the EE subscale, 55% (n = 55, 95%CI: 44.8%-64.9%) in the DP subscale, and 45% (n = 45, 95% CI: 35.1%-55.2%) in the AP subscale. Only 14% (n = 14) of the participants had no high level of burnout in any of the subscales. Participants with a high level of burnout in one subscale constituted 29% (n = 29). Those in two subscales represented 34% (n = 34), and those in all three subscales made up 23% (n = 23). The prevalence of burnout was 86% (n = 86, 95% CI: 77.3%-91.9%). A significant positive, fair correlation existed between the AP score and the monthly income (tau = 0.211, p = 0.010). Burnout was significantly associated with younger age (p = 0.002) and lower monthly income (p = 0.007).

Conclusion

The prevalence of burnout among Saudi ED physicians is high, particularly among young residents and those with lower monthly incomes. Health authorities should establish a strategy to reduce burnout among emergency physicians, particularly among younger residents.

## Introduction

Burnout is defined in the 11th Revision of the International Classification of Diseases as “a syndrome conceptualized as resulting from chronic workplace stress that has not been successfully managed. It is characterized by three dimensions: feelings of energy depletion or exhaustion; increased mental distance from one’s job, or feelings of negativism or cynicism related to one's job; and reduced professional efficacy” [1].

Burnout syndrome has become an increasingly critical concern in healthcare settings worldwide, particularly among physicians who are consistently exposed to high levels of occupational stress and emotional demands. According to Maslach and Jackson [2], burnout consists of three core dimensions: emotional exhaustion (EE), depersonalization (DP), and reduced personal accomplishment (AP). EE reflects feelings of being emotionally depleted and overextended and is considered the most prominent and debilitating core dimension of burnout. DP involves a detached and impersonal response toward patients, which often emerges as a coping mechanism but may exacerbate emotional fatigue. AP relates to an individual’s sense of competence and achievement in their work, potentially serving as a buffer against burnout; its precise role remains complex and context-dependent [2,3].

Physicians who have burnout can experience symptoms of moodiness, depression, and loss of interest in occupational or personal life activities [4]. Those health effects subsequently may interfere with the physician's ability to present quality healthcare by weakening clinical judgment [5], consequently resulting in reduced patient satisfaction and increased likelihood of committing medical errors. In severe cases, burnout may lead to suicide ideation, depression, and addiction [6].

In a study examining burnout among 7,288 physicians in the United States, the highest rates of burnout were identified among emergency department (ED) physicians and general internal medicine practitioners compared to other medical professionals [7]. In addition, more than 60% of ED physicians were found to have moderate to high burnout levels compared to 38% of physicians [8]. Emergency physicians are especially vulnerable to burnout due to the high-intensity nature of their work environment, which often involves long shifts, unpredictable caseloads, life-and-death decision-making, irregular work hours, and emotional exposure to suffering and trauma [9-12].

In Saudi Arabia, recent healthcare reforms and increasing patient demands have further intensified the workload of EDs, underscoring the urgency of understanding and mitigating burnout in this context. Reports have shown high levels of burnout among Saudi emergency medicine residents, emphasizing the urgent need for institutional interventions [10]. Cultural expectations, workload demands, and the evolving healthcare system in Saudi Arabia further complicate physicians' work-life balance and mental health.

Despite the growing attention to physician burnout globally, empirical studies focusing on emergency physicians in the Gulf region, and specifically in Jeddah, remain limited. Therefore, this study seeks to address this gap by determining the prevalence and risk factors of burnout syndrome among the ED physicians working in the governmental general hospitals in Jeddah, Saudi Arabia. The research aims to provide valuable insights for developing a support system for burnout among ED physicians and to present recommendations in the form of new strategies for reducing burnout and improving the well-being of physicians in Jeddah.

## Materials and methods

Ethical considerations

The study was conducted in accordance with ethical standards for research involving human participants. Participation was voluntary, and no identifiable personal information was collected. The study protocol, including informed consent procedures and data management strategies, was reviewed and approved by the Institutional Review Board, Directorate of Health Affairs in Jeddah, Ministry of Health, Saudi Arabia (A02141).

Study design and settings

This cross-sectional study included emergency physicians in governmental hospitals in Jeddah, Saudi Arabia.

Inclusion criteria

Eligible participants were Saudi physicians currently working in EDs at governmental hospitals in Jeddah who voluntarily consented to participate in the study. The hospitals included King Abdullah Medical Complex, King Fahad General Hospital, East Jeddah General Hospital, King Abdulaziz Hospital, Al-Thager General Hospital, Rabigh General Hospital, Al-Lith General Hospital, and Adham General Hospital.

Exclusion criteria

Excluded hospitals were the National Guard Hospital, King Fahad Armed Forces Hospital, specialized hospitals like Eye Hospital, Al Aziziyah Children Hospital, Mental Health Hospital in Jeddah, and private hospitals. Non-Saudi ED physicians, physicians from other specialties who are taking external rotations in ED, physicians from other specialties, medical interns, and trainees in ED were excluded.

Sampling

Participants were recruited using a non-probability, purposive sampling method. The online survey was distributed through professional networks, hospital administrators, and physician groups between the start of March to the end of May 2025. The total number of emergency physicians working in all governmental hospitals in Jeddah was 253, but only 227 were recorded visiting the survey, and a total of 100 emergency physicians submitted their responses to the survey. The response rate was approximately 44%.

Data collection tool

A self-administered, structured questionnaire was used for data collection, comprising three main sections (Appendices). The first section included an introduction outlining the study’s purpose, instructions for completing the questionnaire, and the informed consent form. The second section collected demographic and occupational information, including gender, age, marital status, number of children, monthly income, job title, hospital affiliation, years of experience, shift type, number of monthly shifts, and average sleep duration. The third section comprised the Maslach Burnout Inventory - Human Services Survey for Medical Personnel (MBI-HSS (MP)) [3]. This validated tool was used in its original form with licensed permission. It includes three subscales: a) EE (nine items; e.g., “I feel emotionally drained from my work”), b) DP (five items; e.g., “I don’t really care what happens to some patients”), and c) AP (eight items; e.g., “I have accomplished many worthwhile things in this job”). Responses were rated on a seven-point Likert scale ranging from 0 ("never") to 6 ("every day"). Previous studies have reported the validity of the MBI-HSS tool across different cultures [13,14]. We used the English version, as physicians in Saudi Arabia are well acquainted with this language and all medical curricula are taught in English.

Data collection procedure

The questionnaire was hosted online via Google Forms (Google, Inc., Mountain View, CA). After obtaining a list of ED physicians' WhatsApp (Meta, Menlo Park, CA) contact numbers from the human resources departments of participating hospitals, coordination was made with the heads of the respective EDs to notify their teams about the study. A personalized WhatsApp message was then sent to each eligible physician, including a brief introduction to the study and a link to the questionnaire. Before participation, all respondents were provided with detailed information about the study’s objectives and procedures. Informed consent was obtained electronically, and only those who consented were allowed to proceed with the questionnaire.

Statistical analysis

All data were exported from Google Forms and analyzed using the R Statistical (R Foundation for Statistical Computing, Vienna, Austria) language version 4.5.0 [15]. The internal consistency of the subscales used in this study was assessed by calculating Cronbach’s alpha coefficients. Alpha values between 0.7 and 0.9 are acceptable [16]. Categorical variables (e.g., job title) were presented as frequencies. The categories that included fewer than five observations were merged with other relevant categories. The association with the studied groups was assessed using Pearson’s chi-square test for independence of observations, Fisher’s exact test (if the expected count <5 in more than 20% of cells), or chi-square test for trend in proportions (if one variable was ordinal and the other is nominal). The distribution of continuous numerical variables was examined using the Shapiro-Wilk test and Q-Q plots. All variables that followed a normal distribution were summarized using the mean, standard deviation, and range. Correlations were assessed using Kendall's tau for all relevant variables except sex, which was assessed using point biserial correlation. Statistical significance was set at p < 0.05.

## Results

The present study included 100 participants. Reliability testing for each subscale showed a Cronbach’s alpha of 0.911 (95% CI: 0.871-0.936) for AP, 0.844 (95% CI: 0.788-0.883) for DP, and 0.955 (95% CI: 0.937-0.968) for EE, indicating good internal consistency within each subscale. The Cronbach’s alpha value for the overall inventory was 0.889 (95% CI: 0.832-0.926).

Most participants were in the age group “25-34 years” (n = 83, 83%), and male physicians (n = 58, 58%) outnumbered females (n = 42, 42%). Regarding marital status, 58% (n = 58) were single, and 42% (n = 42) were married. Most participants had no children (75%, n = 75), and 7% (n = 7) had more than two children. Monthly income ranged between 10,000 SAR and 20,000 SAR (approximately equivalent to 2664 to 5329 USD) in 63% (n = 63) of participants, while 30% (n = 30) had a higher income, reaching 30,000 SAR per month (approximately equivalent to >2664 to 7994 USD). Meanwhile, 7% (n = 7) had a monthly income exceeding 30000 SAR (exceeding 7994 USD; Table 1).

**Table 1:** Participants’ socioeconomic characteristics All variables expressed as counts and percentages.

Characteristic	All participants N = 100
Age (years)	
25-34	83 (83.0%)
35-44	11 (11.0%)
45-54	6 (6.0%)
Gender	
Female	42 (42.0%)
Male	58 (58.0%)
Marital status	
Single	58 (58.0%)
Married	42 (42.0%)
Number of children	
None	75 (75.0%)
1	8 (8.0%)
2	10 (10.0%)
3 or more	7 (7.0%)
Income per month	
10000 to 20000 SAR (2664 to 5329 USD)	63 (63.0%)
> 20000 to 30000 SAR (>2664 to 7994 USD)	30 (30.0%)
> 30000 SAR (7994 USD)	7 (7.0%)

The majority of participants were residents or general practitioners (81%, n = 81), while specialists and consultants accounted for 12% (n = 12) and 7% (n = 7), respectively. As for the work experience, 26% (n = 26) had an experience of less than one year, and 47% (n = 47) had an experience between one and five years. One-quarter worked at King Fahad General Hospital, while 24% (n = 24) worked at King Abdullah Medical Complex and 16% (n = 16) at East Jeddah General Hospital. The majority of physicians had rotational shifts (89%, n = 89), which means that they had alternating day, evening, and night shifts according to the hospital’s schedule. The average number of shifts per month ranged between 16 and 20 (59%, n = 59), while 32% (n = 32) had 15 shifts or fewer per month. The average sleep duration for 65% of the studied physicians was six hours or less (n = 65; Table 2).

**Table 2:** Participants’ work characteristics All variables expressed as counts and percentages.

Characteristic	All participants N = 100
Job title	
Resident/general practitioner	81 (81.0%)
Specialist	12 (12.0%)
Consultant	7 (7.0%)
Work experience in years	
Less than one year	26 (26.0%)
One to less than five years	47 (47.0%)
Five to less than 10 years	16 (16.0%)
10 to less than 15 years	6 (6.0%)
15 years or more	5 (5.0%)
What hospital are you working at?	
King Fahad General Hospital	25 (25.0%)
King Abdullah Medical Complex	24 (24.0%)
East Jeddah General Hospital	16 (16.0%)
Al-Thager General Hospital	13 (13.0%)
King Abdulaziz Hospital	13 (13.0%)
Rabigh General Hospital	8 (8.0%)
Al-Lith General Hospital	1 (1.0%)
Shift pattern	
Day shifts only	8 (8.0%)
Evening/night shifts only	3 (3.0%)
Rotational shifts	89 (89.0%)
Average number of shifts per month	
15 or less	32 (32.0%)
16-20	59 (59.0%)
More than 20	9 (9.0%)
Sleep duration on workdays (average)	
Six hours or less	65 (65.0%)
>6 hours	35 (35.0%)

**Figure 1:**
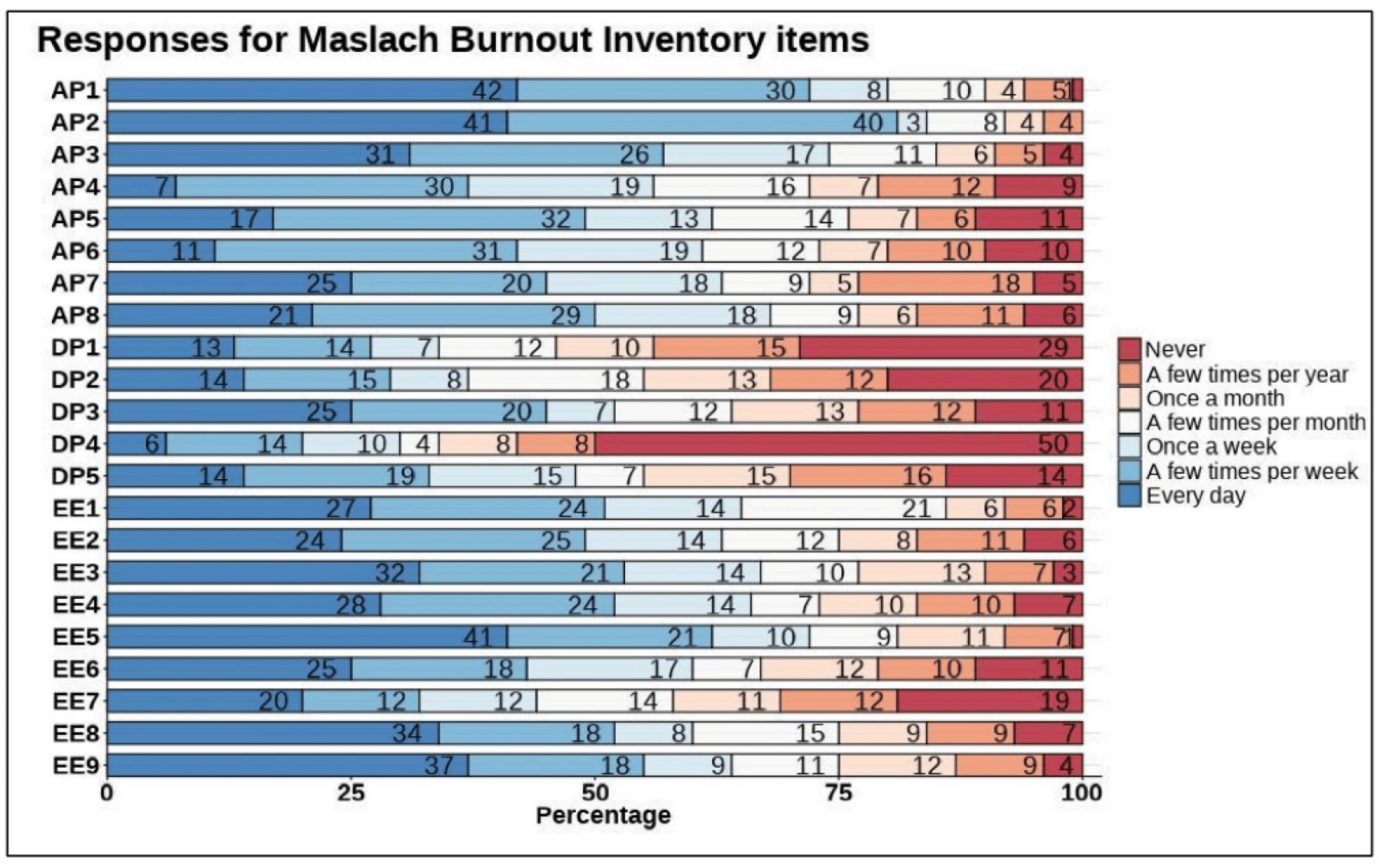
Participants’ responses to the Maslach Burnout Inventory AP: personal accomplishment; DP: depersonalization; EE: emotional exhaustion

The participants’ responses to the three subscales of the Maslach Burnout Inventory (MBI) were recorded. Regarding the AP subscale, the results showed that most physicians had high levels of professional efficacy and fulfillment. The items with the highest grading included AP1 (“I can easily understand how my patients feel about things"), AP2 ("I deal very effectively with the problems of my patients"), and AP3 ("I feel I am positively influencing other people's lives through my work"), which scored “every day” in 42% (n = 42), 41% (n = 41), and 31% (n = 31) of included physicians, respectively (Figure 1).

As for the DP subscale, some items raised concerns. Considerable percentage of physicians displayed high grades of DP in the items DP3 ("I worry that this job is hardening me emotionally"), DP5 ("I feel patients blame me for some of their problems"), DP2 ("I have become more callous toward people since I took this job"), and DP1 ("I feel I treat some patients as if they were impersonal objects"), where 45% (n = 45), 33% (n = 33), 29% (n = 29), and 27% (n = 27) of physicians responded with “a few times per week” or “everyday” (Figure 1). 

On analysis of the EE subscale, we found that a considerable percentage of physicians demonstrated EE, especially in items EE5 ("I feel I am working too hard on my job"), EE9 ("I feel fatigued when I get up in the morning and have to face another day on the job"), EE3 ("I feel burned out from my work"), EE4 ("I feel frustrated by my job"), EE8 ("I feel used up at the end of the workday"), EE1 ("I feel emotionally drained from my work"), and EE2 ("Working with people all day is really a strain for me"), with 62% (n = 62), 55% (n = 55), 53% (n = 53), 52% (n = 52), 52% (n = 52), 51% (n = 51), and 49% (n = 49), respectively, scored “a few times per week” or “everyday” (Figure 1).

The scores for each subscale were calculated, and the levels of burnout in each subscale were accordingly derived. As regards the EE subscale, 66% (n = 66) of included physicians had a high level of burnout (95% CI: 55.8% to 75%). The DP subscale showed a high burnout level in 55% (n = 55) (95% CI: 44.8% to 64.9%), while the prevalence was 45% (n = 45) in the personal achievement subscale (95% CI: 35.1% to 55.2%). Only 14% (n = 14) of the participants had no high level of burnout in any of the subscales. Participants with a high level of burnout in one subscale constituted 29% (n = 29). Those in two subscales represented 34% (n = 34), and those in all three subscales made up 23% (n = 23). For the purposes of this study, we calculated the prevalence of burnout as the percentage of physicians with high-level burnout in at least one subscale. The prevalence of burnout was 86% (n = 86) (95% CI: 77.3% to 91.9%). Using the finite population correction score (for a total population of 253 physicians), the prevalence was estimated as 86% (95% CI: 79.37 to 89.97%; Table 3).

**Table 3:** Burnout scores, levels, and prevalence in the studied participants AP: personal accomplishment; CI: confidence interval; DP: depersonalization; EE: emotional exhaustion; SD: standard deviation

Characteristic	All participants N = 100	95% CI
AP score, mean ± SD (range)	32.5 ± 11.0 (5.0-48.0)	30.3-34.7
DP score, mean ± SD (range)	13.7 ± 8.4 (0.0-30.0)	12.0-15.4
EE score, mean ± SD (range)	35.4 ± 14.8 (0.0-54.0)	32.5-38.4
EE levels, n (%)		
High-level burnout	66 (66.0%)	55.8%-75.0%
Low-level burnout	14 (14.0%)	8.1%-22.7%
Moderate burnout	20 (20.0%)	12.9%-29.4%
DP levels, n (%)		
High-level burnout	55 (55.0%)	44.8%-64.9%
Low-level burnout	22 (22.0%)	14.6%-31.6%
Moderate burnout	23 (23.0%)	15.4%-32.7%
AP levels, n (%)		
High-level burnout	45 (45.0%)	35.1%-55.2%
Low-level burnout	36 (36.0%)	26.8%-46.3%
Moderate burnout	19 (19.0%)	12.1%-28.3%
Scales with high-level burnout, n (%)		
None	14 (14.0%)	8.1%-22.7%
One subscale	29 (29.0%)	20.6%-39.1%
Two subscales	34 (34.0%)	25.0%-44.2%
All three subscales	23 (23.0%)	15.4%-32.7%
Burnout, n (%)		
Burnout	86 (86.0%)	77.3%-91.9%
No burnout	14 (14.0%)	8.1%-22.7%

Next, we assessed the correlation between the scores of each of the subscales and the participants’ characteristics. A significant positive, fair correlation existed between the AP score and the monthly income, indicating an increase in the experienced personal achievement with the increase in income (tau = 0.211, p = 0.010). No other significant correlations were detected (all p-values > 0.05; Table 4).

**Table 4:** Correlation between the scores of the Maslach Burnout Inventory subscales and participants’ characteristics ^a^Correlation coefficient (Kendall's tau for all except sex, which is point biserial). *Significant at p < 0.05. AP: personal accomplishment; DP: depersonalization; EE: emotional exhaustion

Tested correlations		Correlation coefficient^a^	p-value
AP score	Age (years)	0.122	0.138
	Gender	0.009	0.927
	Number of children	0.084	0.295
	Income per month	0.211	0.010*
	Work experience in years	0.111	0.152
	Average number of shifts per month	-0.105	0.197
	Sleep duration on workdays (average)	0.007	0.937
DP score	Age (years)	-0.067	0.418
	Gender	0.05	0.624
	Number of children	-0.07	0.387
	Income per month	-0.119	0.144
	Work experience in years	0.015	0.846
	Average number of shifts per month	-0.014	0.864
	Sleep duration on workdays (average)	-0.045	0.595
EE score	Age (years)	-0.072	0.382
	Gender	-0.104	0.303
	Number of children	-0.11	0.17
	Income per month	-0.087	0.284
	Work experience in years	-0.058	0.457
	Average number of shifts per month	0.099	0.22
	Sleep duration on workdays (average)	-0.037	0.656

Burnout was significantly associated with younger age (p = 0.002), as 90% (n = 75) and 73% (n = 8) of those aged “25-34 years” and “35-44 years” had burnout. In addition, burnout was significantly associated with lower monthly income (p = 0.007). Burnout was detected in 92% (n = 58) of those earning 10,000 to 20,000 RAS and in 80% (n = 24) of those earning >20,000 to 30,000 SAR. No significant association was found between burnout and each of gender (p = 0.944), marital status (p = 0.216), number of children (p = 0.112), job title (p = 0.277), work experience (p = 0.067), shift pattern (p = 0.293), number of shifts per month (p = 0.180), or sleep duration (p > 0.999; Table 5).

**Table 5:** Comparisons of participants’ characteristics between physicians with and without burnout ^a^Chi-squared test for trend in proportions. ^b^Pearson’s chi-squared test. ^c^Fisher’s exact test. *Significant at p < 0.05.

Characteristic	Burnout N = 86	No burnout N = 14	Test statistic	p-value
Age (years)			9.34	0.002^*a^
25-34	75 (90%)	8 (9.6%)	-	-
35-44	8 (73%)	3 (27%)	-	-
45-54	3 (50%)	3 (50%)	-	-
Gender			0.005	0.944^b^
Female	36 (86%)	6 (14%)	-	-
Male	50 (86%)	8 (14%)	-	-
Income per month (SAR)			7.331	0.007^*a^
10000 to 20000	58 (92%)	5 (7.9%)	-	-
> 20000 to 30000	24 (80%)	6 (20%)	-	-
> 30000	4 (57%)	3 (43%)	-	-
Marital status			1.532	0.216^b^
Single	52 (90%)	6 (10%)	-	-
Married	34 (81%)	8 (19%)	-	-
Number of children			2.522	0.112^a^
None	66 (88%)	9 (12%)	-	-
One	8 (100%)	0 (0%)	-	-
Two	7 (70%)	3 (30%)	-	-
Three or more	5 (71%)	2 (29%)	-	-
Job title				0.277^c^
Resident/general practitioner	71 (88%)	10 (12%)	-	-
Specialist	10 (83%)	2 (17%)	-	-
Consultant	5 (71%)	2 (29%)	-	-
Work experience in years			3.367	0.067^a^
Less than one year	22 (85%)	4 (15%)	-	-
One to less than five years	43 (91%)	4 (8.5%)	-	-
Five to less than 10 years	15 (94%)	1 (6.3%)	-	-
10 to less than 15 years	3 (50%)	3 (50%)	-	-
15 years or more	3 (60%)	2 (40%)	-	-
Shift pattern				0.293^c^
Day shifts only	8 (100%)	0 (0%)	-	-
Evening/night shifts only	2 (67%)	1 (33%)	-	-
Rotational shifts	76 (85%)	13 (15%)	-	-
Average number of shifts per month			1.798	0.180^a^
15 or less	26 (81%)	6 (19%)	-	-
16-20	51 (86%)	8 (14%)	-	-
More than 20	9 (100%)	0 (0%)	-	-
Sleep duration on workdays (average)				>0.999^c^
Six hours or less	56 (86%)	9 (14%)	-	-
>6 hours	30 (86%)	5 (14%)	-	-

## Discussion

ED physicians are vulnerable to burnout due to the high-intensity nature of their work environment [9-12]. However, studies focusing on emergency physicians in Saudi Arabia remain limited. The present study aimed to determine the prevalence and risk factors of burnout syndrome among the ED physicians working in the governmental general hospitals in Jeddah, Saudi Arabia.

The response rate in this study was rather low, approximately 44%, but it accords with response rates reported among physicians to web-based surveys. Previous studies have reported low survey response rates that range between 29% and 46% among physicians from different specialties [17,18]. The low response rate in the present study may be attributable to the high workload and time restrictions experienced by ED physicians.

Most participants were in the age group “25-34 years.” This accords with the general concept that emergency medicine is regarded as a young person’s specialty, owing to the high-intensity work environment [19]. However, since the current study did not utilize random sampling, there is potential selection bias, with younger ED physicians possibly being more likely to participate than their older counterparts. This may be attributable to younger physicians’ greater awareness of burnout syndrome and a higher willingness to report their experiences without concern for stigmatization compared to older physicians [20].

In the current study, the participants showed high grades of DP as observed from their scores on the DP subscale of MBI. The DP subscale depicts cynicism, which refers to appearing colder or more detached than regular people who are coping with work stresses [21]. High DP levels may be attributed to hostile criticism by seniors and patients, short temper, and suffering from sleep disturbances [22]. In addition, ED physicians encounter death daily due to the critical nature of many emergency conditions, and are usually the ones charged with communicating the unfortunate news to the deceased’s friends and family. Over time, this constant exposure to such trauma can lead to emotional detachment [19].

We found that a considerable percentage of the participants suffered from EE as demonstrated by their answers to the EE subscale. More than half of the participants responded with high scores to feeling they are working too hard, fatigued in the morning, burned out from work, frustrated by their job, used up at the end of the workday, and emotionally drained from their work. High exhaustion scores indicate that participants feel overworked, leading to depleted mental and physical resources [21]. High levels of EE can be attributed to work overload, an inability to balance work and personal life, fatigue, and the fear of making mistakes, which is often referred to as second victim syndrome [22].

In the present study, most ED physicians showed high levels of professional efficacy and fulfillment as measured by the AP subscale of MBI, particularly understanding patients’ feelings, dealing effectively with patients’ problems, and feeling positively influenced by other people's lives through work. The high levels of professional efficacy and fulfillment of the emergency physicians in this study likely reflect the combined influence of ongoing healthcare reforms under Vision 2030, such as integrated health services, expanded digital health services, mobile units, and a focus on patient-centered care, and cultural factors emphasizing interpersonal respect and social responsibility. We believe all these factors have contributed to the satisfactory physicians’ ability to understand and positively impact patients’ lives. Targeted interventions should sustain physicians' well-being and commitment within the evolving Saudi medical system [23].

In this study, high scores on the AP subscale suggest feeling higher work success and feeling better AP [21]. This finding is supported by the findings of Shanafelt et al. [7], who assessed burnout and work satisfaction in more than 7,000 physicians in the United States and reported that more than half of ED physicians were satisfied with their work-life balance, despite having the highest burnout rate among the different specialties.

The prevalence of high levels of burnout was 66%, 55%, and 45% according to the EE, DP, and AP subscales, respectively. Participants with a high level of burnout in one subscale constituted 29%. Those in two subscales represented 34%, and those in all three subscales made up 23%. Only 14% of the participants did not exhibit high burnout levels in any of the three subscales. Our findings partially agree with those of previous similar studies, which reported higher or lower rates in some subscales, while they accorded with our findings in the others.

For instance, Schooley et al. [24] compared burnout amongst ED staff and found that 71.1% and 78.94% of physicians had high levels of EE and DP, respectively, while 28.9% had a low level of AP score.

In addition, Wilson et al. [22] assessed burnout in 105 ED professionals in four tertiary care hospitals from South India. They found that the prevalence of moderate to severe burnout was 64.8%, 71.4% and 73.3% in the EE, DP, and AP subscales, respectively. The higher rates in the DP and AP subscales may be due to the combination of moderate and high levels of burnout in their study.

In the Aseer Region, Southwestern Saudi Arabia, Alqahtani et al. [25] surveyed 95 ED physicians and reported a high EE level in 81.1%, while high DP and low AP levels were reported in 24.2% and 27.4%, respectively.

Moreover, Alsaawi et al. [26] evaluated burnout in 303 ED physicians attending a large national emergency medicine conference in Saudi Arabia and found that the prevalence of high risk of burnout was 35.8%, 50.9%, and 40.4% based on EE, DP, and AP subscales. They also reported that 72.1% had high risk in at least one subscale, whereas 56.3% showed high risk in EE or DP domains, and 13.4% exhibited high risk scores in the three domains.

A recent meta-analysis by Alanazy et al. [19] reported also lower pooled prevalence rates, where that of high EE score was 39% (95% CI: 28-49%), high DP was 43% (95% CI: 36-50%), and low AP was 36% (95% CI: 28-44%).

In the current study, the overall prevalence of burnout was 86% (n = 86, 95% CI: 77.3% to 91.9%). The overall prevalence of burnout in the medical literature showed wide variations, ranging from 60% to 77.8% among emergency trainees [22,27-30].

The reported high rates of burnout among ED physicians could be explained by the hectic nature of the ED where physicians are required to make life-saving medical decisions in a very limited time window with insufficient information, and are continually exposed to the stresses of receiving critical cases, dealing with agitated and emotionally depressed families, and conveying bad news to patients’ friends and relations [8,22]. However, the relatively small sample size in this study warrants cautious interpretation of this rate, and further future studies should plan for a priori sample size calculation to confirm the true prevalence of burnout among emergency physicians in Saudi hospitals.

On the other hand, much lower rates were also reported by studies from Saudi Arabia, as well as by a meta-analysis encompassing studies from different countries. Alqahtani et al. [25] found that the overall burnout rate was 18.9% based on the three subscales. In addition, Alsaawi et al. [26] found that 13.4% were in the high-risk group in all three burnout domains. Also, a recent meta-analysis [19] that comprised 11 studies reported that the rate of burnout in ED physicians was 51% (95% CI: 28-74%). The difference in the reported prevalence rates of burnout and its subscales across the aforementioned studies can be attributed to variations in the work environment of the healthcare system, patients’ attitudes, as well as differences in the study design, the utilized assessment tools, and the definitions adopted for defining burnout.

In this study, we assessed the correlation between the scores of each of the subscales and the participants’ characteristics.

We found that burnout was significantly associated with younger age (p = 0.002), particularly among those between 25 and 34 years. Likewise, Alqahtani et al. [25] reported that younger ED staff may be more vulnerable to feelings of low AP compared to older physicians. Similarly, Alsaawi et al. [26] found a negative correlation between the physician’s age and DP scores (rho = −0.13; p = 0.03). This is largely due to their lower salaries and the challenges they face in developing professional skills and knowledge. Moreover, younger ED physicians may be at a higher risk of making medical errors due to their limited experience, which can increase their stress and overall burden.

Among the studied sample, burnout was significantly associated with lower monthly income (p = 0.006), which was also depicted in assessing the correlation between the AP score and the monthly income (tau = 0.210, p = 0.010). This finding indicates an increase in the experienced personal achievement with the increase in income, underscoring salary as one of the important factors contributing to AP and how it can buffer some of the stresses experienced by ED physicians.

We found no significant association between burnout and the physicians’ gender (p = 0.944), marital status (p = p = 0.102), or number of children (p = 0.059). Our results partially agree with those of Baruah et al. [31], who reported a lack of significant association between the demographic variables and EE or DP, while having children was significantly associated with low burnout level in AP. On the other hand, Abdo et al. [32] reported significantly different rates in Egypt. Out of 266 ED physicians, 39.7% had high EE, 99.2% showed low AP, and 22.6% expressed high DP.

Regarding work-related factors, including job title, work experience, shift pattern, number of shifts per month, and sleep duration, we did not find a significant association with burnout. Likewise, other previous studies reported a lack of significant association between burnout and years of experience [25,26]. On the other hand, other previous studies reported that years of experience and work burden, and work activities were significant predictors of burnout [31-33], but these studies included other ED personnel besides the physicians.

While the present study threw light on some of the less explored aspects of burnout among medical professionals, namely, ED physicians, the study was prone to some limitations. First, being a cross-sectional study, causality between burnout and the assessed factors cannot be established. Longitudinal studies are warranted to assess how the different components of burnout undergo alterations over time. Second, we included ED physicians working at governmental hospitals in one city, which can limit the generalizability of our findings to other health sectors or geographical regions. Physicians practicing in private hospitals were not included in the study due to potential concerns regarding the expression of candid opinions, as fear of workplace repercussions may have inhibited their willingness to participate openly in the survey. Third, the sample size was not determined based on prior statistical calculations and was relatively small despite our efforts to remind the physicians to participate and our attempts to reach all ED physicians in Jeddah’s governmental hospitals. The small sample size may limit the study’s statistical power to detect significant effects. Consequently, the findings should be interpreted cautiously, and the results may not be generalizable to broader populations. Future studies with appropriately powered sample sizes are warranted to confirm these results. Fourth, although the MBI is a validated tool, its self-reported nature may introduce recall bias. Also, participants may respond in a socially desirable way, which could affect the accuracy of their responses.

## Conclusions

The present study underscored the high prevalence of burnout among Saudi ED physicians, particularly among young residents and those with lower monthly income. Health authorities should establish a strategy to reduce burnout among emergency physicians, particularly among younger residents. For addressing this problem, collaborative efforts from the concerned authorities should focus on institutional reform by establishing rules for the maximum number of shift hours and the number of shifts per week to reduce the workload and help ED physicians have a balance between work hours, time needed to promote their postgraduate studies, and personal life. Moreover, we recommend establishing training programs for emotional skills to equip ED physicians with the necessary skills to cope with stress, achieve work-personal life balance, follow a healthy lifestyle, and avoid feelings of burnout. Future studies should preferably be longitudinal in design and explore other potentially influencing variables, such as leadership support and coping strategies. Qualitative studies are also required for a thorough exploration of the experiences of ED physicians and how they respond to EE. However, these conclusions should be interpreted with caution, given the study’s relatively small sample size, cross-sectional design, and restriction to a single geographic region, which may limit the generalizability of the findings.

## Figures and Tables

**Figure 2:**
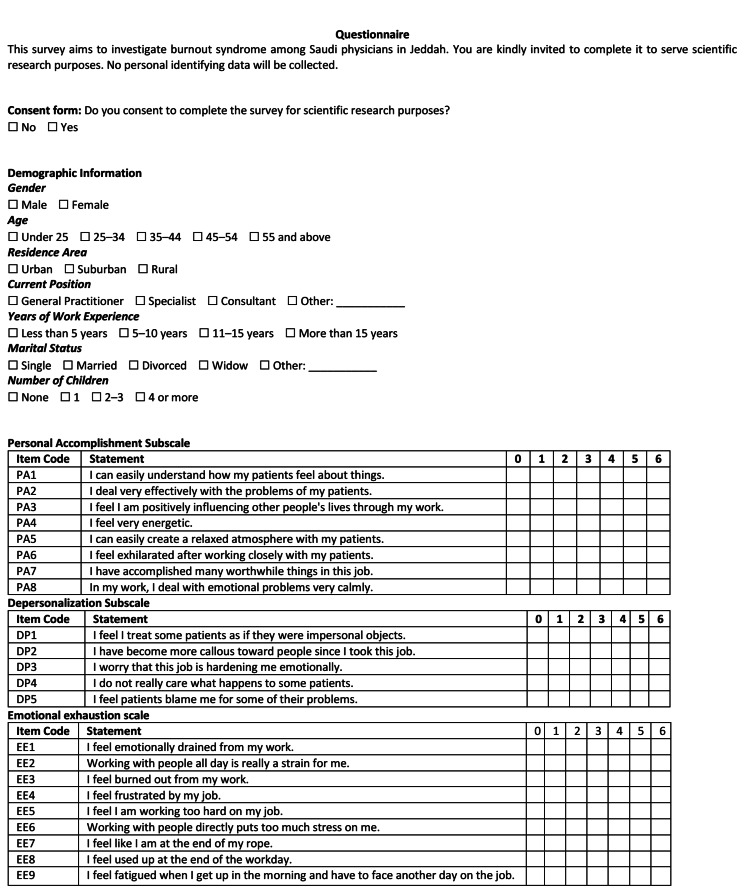
Data collection tool (questionnaire)
